# Leucocyte Telomere Shortening in relation to Newly Diagnosed Type 2 Diabetic Patients with Depression

**DOI:** 10.1155/2014/673959

**Published:** 2014-04-29

**Authors:** Zhelong Liu, Jianhua Zhang, Jiangtao Yan, Yuping Wang, Yongsheng Li

**Affiliations:** ^1^Division of Endocrinology, Tongji Hospital, Tongji Medical College, Huazhong University of Science and Technology, Jiefang Avenue 1095, Wuhan 430030, China; ^2^Division of Cardiology, Tongji Hospital, Tongji Medical College, Huazhong University of Science and Technology, Jiefang Avenue 1095, Wuhan 430030, China; ^3^Division of Emergency Internal Medicine, Tongji Hospital, Tongji Medical College, Huazhong University of Science and Technology, Wuhan 430030, China

## Abstract

The goal of this study is to investigate the association between oxidative stress and telomere length shortening in the comorbid depression and diabetes. Therefore, 71 patients with newly diagnosed type 2 diabetes (T2D) and 52 subjects with normal glycemic level (control, Ctrl) were enrolled. Depressive status was identified with the Depression Subscale of Hospital Anxiety and Depression Scale (HADS-D). Leukocyte telomere length ratio (T/S ratio) was determined with quantitative PCR. Oxidative stress status was evaluated with 8-hydroxy-desoxyguanosine (8-OHdG) assay kit. Some other biochemical blood testing was also performed. The data showed that T2D patients had higher proportion of depression evaluated by the HADS-D (*x* ^2^ = 4.196, *P* = 0.041). T/S ratio was significantly negatively correlated with 8-OHdG, HADS-D, age, HbA1c, FPG, and HOMA-IR. In addition, HADS-D was significantly positively correlated with HbA1c, FPG, HOMA-IR, and 8-OHdG. Both HADS-D and 8-OHdG were the major independent predictors for T/S ratio. This study indicates that oxidative stress contributes to both telomere length shortening and depression development in newly diagnosed type 2 diabetic patients, while in depression status, some other mechanisms besides oxidative stress may also affect the telomere length.

## 1. Introduction

Telomeres are tandem repeats of DNA sequence, TTAGGG at the end of eukaryotic chromosomes [1]. The important function of telomere is to protect the genomic DNA from being degenerating and maintain the genomic stability [2]. The telomere decreases with repeated cell division. When the shortened length gets to some exact extent, the cell develops to senescence. Oxidative stress is considered to be tightly related to the procedure of telomere decrease as it can induce the strand breaks of telomeric DNA [3, 4].

Recently increasing evidence showed the association between the shortening of leucocyte telomere length and several age-related diseases, including type 2 diabetes [5–7]. Type 2 diabetes (T2D), characterized with the clinical chronic hyperglycemia and insulin resistance, is nowadays one of the most threatening problems to the global public health. Although it has not been fully comprehended, more and more evidence shows that both oxidative stress and cell premature senescence may take an important part in the mechanism of T2D [5]. Some previous studies including our work suggested a probable relationship between oxidative stress and the leucocyte telomere length shortening in diabetic patients [8, 9].

The association of psychological stress and illness with telomere length change has also been reported lately [10, 11]. Depression, one of the most common forms of psychological disorders, often cooccurs with type 2 diabetes [12]. Diabetic patients with depression have higher HbA1c levels and poorer glycemic control [13]. They may have less physical exercises and be less compliant to take healthy diet and antidiabetic regimen [14]. What is more, it is verified that diabetic patients with depression have higher mortality rates due to myocardial infarction [15] and a latest systematic review shows that depression is associated with almost 1.5-fold increase risk of mortality in people with diabetes [16]. However, the mechanism of the combination and linkage of depression and diabetes is still unknown.

Although the telomere length decrease is identified in diabetic or depressive patients, respectively, there is no research work reported in the population of cooccurring depression and diabetes till now. Therefore, the aim of our study was to investigate the association between oxidative stress and telomere length shortening in the comorbid depression and diabetes.

## 2. Research Design and Methods

### 2.1. Patients and Controls

A total of 71 patients with newly diagnosed type 2 diabetes (T2D) (male 40/female 31) were recruited from the Division of Endocrinology, Tongji Hospital, Tongji Medical College, Huazhong University of Science and Technology, Wuhan, Hubei, China, between January 2011 and June 2012. The subjects were questioned about their medical history and family history. The enrolled subjects were diagnosed with T2D no more than 1 month and had not received any antidiabetic agents yet. The diagnosis of diabetes was in accordance with World Health Organization criteria (fasting plasma glucose ≥ 7 mmol/L or 2-hour plasma glucose ≥ 11.1 mmol/L) [17]. 52 subjects with normal glycemic level were enrolled in control group (Ctrl group) (male 30/female 22). The individuals with pregnancy, acute inflammation, communicable diseases, cancer, stroke, severe cardiovascular disease, Alzheimer's disease, dementia, or severe cognitive disorders were excluded. In this study, the definition of drinkers was those who consumed liquor within the last 30 days and the average pure alcohol intake ≥10 g per week. This study was carried out in accordance with the principle of Helsinki Declaration and approved by the local ethical committee. All participants gave written informed consent to participate in this study.

### 2.2. Assessment of Anthropometric Data

All subjects took physical examination by a physician. Blood pressure was measured in the sitting position after resting for 10 min. Waist circumference was measured midway between the lowest rib and the iliac crest in the upright standing position. Hip circumference was measured at the greater trochanter.

### 2.3. Assessment of Depression

For screening of depression, we used the Depression Subscale of Hospital Anxiety and Depression Scale (HADS-D), which consists of 7 questions [18]. All the subjects completed the HADS-D separately, without any interaction with research staff. Depression was identified as the score ≥10.

### 2.4. Blood Samples Collection and Laboratory Measurement

Participants were fasted overnight for 10 hours and had blood samples drawn from an antecubital vein, then immediately aliquoted into cryotubes as plasma, buffy coat, and red blood cells. Fasting plasma glucose (FPG) was measured using glucose oxidase method (AVE 2852 half auto biochemical analyzer), fasting insulin (FIN) was measured using electrochemiluminescence assay (Elecsys 2010, Roche Instrument Center AG), and HbA1c was measured using high pressure liquid chromatography (variant II, Bio-Rad). Peripheral insulin resistance was estimated by homeostasis model assessment (HOMA-IR = FIN × FPG/22.5). Serum total cholesterol (TC), triacylglycerols (TG), high-density lipoprotein cholesterol (HDL-C), and low-density lipoprotein cholesterol (LDL-C) levels were measured using enzymatic method performed on clinical chemistry analyzer (Roche/Hitachi MODULAR analyzer).

#### 2.4.1. Measurement of Relative Telomere Length

All buffy-coat cryotubes were stored in freezers at −80°C. Genomic DNA was extracted from peripheral white blood cells using the QG-Mini80 workflow with DB-S kit (Fujifilm Corporation, Tokyo, Japan) as instructed.

Telomere length ratio (T/S ration) was measured using a quantitative PCR-based technique [19]. In this method, the ratio of the telomere repeat copy number (T) and single-copy gene number (S) was compared for each sample. Reactions for DNA samples were run in 7 *μ*L reaction volumes with ABI-7900 HT real-time thermal cycler (Applied Biosystems).

The primers are as follows.

 Telomere-F 5′-CGGTTTGTTTGGGTTTGGGTTTGGGTTTGGGTTTGGGTT-3′.
 Telomere-R 5′-GGCTTGCCTTACCCTTACCCTTACCCTTACCCTTACCCT-3′.
 
*β*-globin-F 5′-GCTTCTGACACAACTGTGTTCACTAGC-3′.
 
*β*-globin-R 5′-CACCAACTTCATCCACGTTCACC-3′.
 
*β*-globin-P FTGCATCTGACTCCTGAGGP.

Cycling conditions of telomere were as follows: 95°C incubation for 10 minutes followed by 35 cycles of 95°C for 15 seconds and 56°C for 1 min. As for the cycling conditions of *β*-globin, 95°C incubation for 10 minutes followed by 40 cycles of 95°C for 15 sec and 56°C for 1 min. The specificity of all of the amplification was determined by melting curve analysis.

#### 2.4.2. Measurement of 8-OHdG

8-OHdG in leucocyte DNA was quantified with OxiSelect oxidative DNA damage ELISA kit (Cell Biolab, Inc, San Diego, USA), which can be used to evaluate the degree of antioxidant stress [9].

### 2.5. Statistical Analysis

Analyses were performed with the SPSS 11.5 (SPSS) statistical package. Significance was defined as the *P* value was less than 0.05. All variables were tested for normal distribution of the data. Data are shown as means ± standard deviation (SD) in a normal distribution, while as median with interquartile range (IQR, 25th~75th percentile) in a nonnormal distribution. Continuous variables differences between two groups were analyzed with Student's test and among multiple groups with ANOVA and for nonparametric data with the Mann-Whitney* U* test or Kruskal-Wallis* H* test, respectively. A chi-square test was utilized for categorical data. To evaluate the reliability of the questionnaire of HADS-D, the internal consistency was used, which was examined with the Cronbach's alpha, and the results >0.7 were judged as adequate. The Spearman correlation analysis was adopted to evaluate the correlation between telomere length and other factors including age, body mass index, waist circumference, waist-to-hip ratio, systolic and diastolic blood pressure, fasting plasma glucose, fasting insulin, HOMA-IR, HbA1c, lipids, 8-OHdG, and HADS-D. The correlation between HADS-D/8-OHdG and other factors was also examined in this way. Stepwise multiple linear regression was applied to determine independent predictors of T/S ratio in T2D group. Candidates for the stepwise multiple regressions were variables that yielded a *P* value of less than 0.15 in the univariate analysis. Then collinear variables were excluded when the Spearman's rank correlation coefficient was more than 0.7. The *P* value was no less than 0.10 for variables entered the regression.

## 3. Result

### 3.1. Main Clinical and Biological Parameters in Subjects

Subjects with and without T2D did not differ significantly in terms of the proportion of smokers, drinkers, and the use of aspirin, statin, and antihypertension drugs. The subjects in T2D group were significantly older than in the control group. The sex ratio was similar in the two groups.

The values of BMI, waist circumference, WHR, the values of systolic blood pressure, diastolic blood pressure, HbA1c, FPG, HOMA-IR, TC, TG, and 8-OHdG were higher in the patients with T2D, while the HDL level was lower in the T2D group. The T/S ratio in the T2D group was significantly shorter than in the control group (Table 1).

T2D group was further divided into two subgroups as with depression [T2D(D+)] and without depression (T2D(D−)); similarly, Ctrl groups also were subclassified as Ctrl(D+) and Ctrl(D−). Among the four subgroups, no significant difference was found in the indexes of age, proportion of male, smokers, drinkers, drugs used, SBP, FIN, HDL, and LDL. Comparing with the T2D(D−) patients, the T2D(D+) ones had higher HADS-D scores and T/S ratio with similar level of BMI, WHR, SBP, DBP, HbA1c, FPG, HOMA-IR, lipids, and 8-OHdG. Comparing with Ctrl(D+) patients, the T2D(D+) ones had higher proportion of hypotensive drugs and higher level of HbA1c, FPG, and TC. No remarkable difference was found in T/S, HOMA-IR, and 8-OHdG. Comparing with the Ctrl(D−) subjects, the Ctrl(D+) ones had higher BMI besides HADS-D scores. The Ctrl(D−) also showed lower level of telomere length (Table 2).

### 3.2. HADS-D Evaluation with Internal Consistency

The chi-square test showed that the T2D patients had higher proportion of depression than control subjects, evaluated by the HADS-D (*x* ^2^ = 4.196, *P* = 0.041) (Table 1). The internal consistency of the HADS-D, as calculated by Cronbach's *α*, was valued as 0.716 in T2D group and 0.730 in Ctrl group, respectively.

### 3.3. Correlation between T/S Ratio and Other Factors in Whole Population

**Figure 1:**
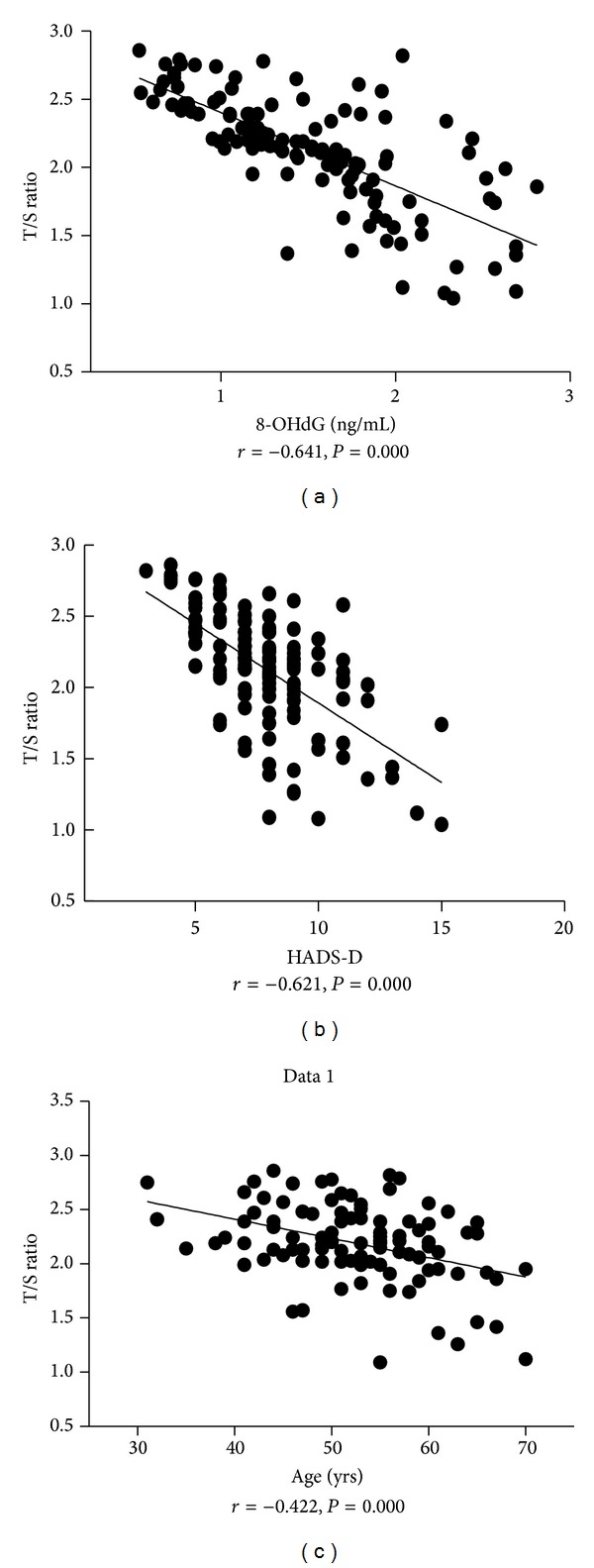
Correlation between T/S ratio and 8-OHdG(a), HADS-D (b), and age (c) in the whole population studied.

The correlation between T/S ratio and other factors was analyzed in the whole population of the two groups (*N* = 123). It was shown that age (*r* = −0.422, *P* = 0.000), HADS-D (*r* = −0.621, *P* = 0.000), HbA1c (*r* = −0.543, *P* = 0.000), FPG (*r* = −0.434, *P* = 0.000), HOMA-IR (*r* = −0.322, *P* = 0.000), 8-OHdG (*r* = −0.641, *P* = 0.000), SBP (*r* = −0.189, *P* = 0.036), DBP (*r* = −0.211, *P* = 0.019), and TG (*r* = −0.2, *P* = 0.026) were significantly negatively correlated with T/S ratio, while FIN, age, BMI, wrist circumference, WHR, TC, TG, and LDL had no significant correlation with T/S ratio. This indicates that the decrease of telomere correlates with depression and oxidative stress besides aging, blood glucose level, and insulin resistance (Figure 1).

### 3.4. Correlation between HADS-D and Other Factors in Whole Population

It was shown that HbA1c (*r* = 0.272, *P* = 0.002), FPG (*r* = 0.239, *P* = 0.008), HOMA-IR (*r* = 0.28, *P* = 0.002), 8-OHdG (*r* = 0.331, *P* = 0.000), and age (*r* = 0.202, *P* = 0.025) were significantly positively correlated with HADS-D, while other indexes had no significant correlation with HADS-D. These results suggested that depression related to hyperglycemia and insulin resistance besides telomere length shortening (as shown in Section 3.3).

### 3.5. Correlation between 8-OHdG and Other Factors in Whole Population

It was shown that HbA1c (*r* = 0.685, *P* = 0.000), FPG (*r* = 0.595, *P* = 0.000), HOMA-IR (*r* = 0.381, *P* = 0.000), age (*r* = 0.267, *P* = 0.003), BMI (*r* = 0.236, *P* = 0.009), wrist circumference (*r* = 0.308, *P* = 0.001), WHR (*r* = 0.247, *P* = 0.006), DBP (*r* = 0.211, *P* = 0.002), TC (*r* = 0.342, *P* = 0.000), and TG (*r* = 0.258, *P* = 0.004) were significantly positively correlated with 8-OHdG, while other indexes had no significant correlation with 8-OHdG. Thereby, oxidative stress relates to telomere length shortening, depression (as shown in Sections 3.2 and 3.3), aging, and insulin resistance with obesity.

### 3.6. Multiple Linear Regression Analysis in T2D Group

Stepwise multiple linear regression in T2D subjects was applied to evaluate independent predictors of T/S ratio. The results showed that both HADS-D and 8-OHdG were the major independent predictors of T/S ratio (*P* = 0.000, 0.001, resp.), thereby indicating that the type 2 diabetic patients with higher scores of HADS-D and in more severe oxidative stress status may have much more shortened telomere length. Other independent predictors included HbA1c, FPG, age, and SBP (Table 3).

## 4. Discussion

The result showed that oxidative stress could play an important role in the mechanism of telomere length shortening. Reactive oxygen species may induce deoxyguanosine conversion to 8-OHdG in the cellular nucleus, which is then released into blood [20]. Therefore, 8-OHdG was utilized as an indicator related to oxidative stress and remarkable negative correlation between 8-OHdG and T/S ratio was observed in the whole population studied, which is consistent with the previous findings [9]. On another side, compared with the diabetic subjects without depression, the diabetic patients with depression had higher level of 8-OHdG in this study. To our knowledge, it is the first time to evaluate the correlation between oxidative stress and depression with the detecting of blood 8-OHdG level and examining of HADS-D score. Furthermore, in both the diabetic and control subjects, the depressive ones had remarkable shorter telomere length than those free of depression, also indicating the relevance between depression and telomere degeneration. Up to now, it is the first time to show the interrelationship of oxidative stress and depression and telomere shortening in type 2 diabetic patients.

Interestingly, in diabetic group, the depressive ones had shorter telomere than those without depression, while their glycemia and 8-OHdG were in similar levels. That may be explained with the bias due to the small sample size in our study. Otherwise, there is the possibility that the depressive status might be more prone to induce telomere shortening throughout another mechanism rather than oxidative stress. Therefore, more studies must be made to answer this confusing question.

Insulin resistance is reported to be in relation to telomere length as the HOMA-IR and BMI are the important indexes for insulin resistance [21–23]. In our study, the T/S ratio is shown to be correlated with HOMA-IR but not with BMI in the whole population studied. In multiple linear regression analysis in T2D group, we failed to identify HOMA-IR to be independent predictor of T/S ratio as the 95% confidence interval for unstandardized coefficient is −0.003, 0.035 and the *P* value is 0.097. This may also be attributed to the small sample size of this study.

The development of depression may be related to many factors. Aged females are reported with higher risk of depression [24, 25]. Life styles such as smoking and drinking and some drugs including aspirin, statins, and antihypertension agents are also indicated to affect the development of depression [26–30]. As for this study, the levels of the sex ratio, age, and the proportion of smokers, drinkers, and the drugs used mentioned above in the depression subgroups between T2D and Ctrl were shown to be similar. Additionally, the duration of diabetes, the insulin injection, and the level of hyperglycemic control may also correlate with depression [16, 31]. For this reason, only patients of newly diagnosed diabetes were included, who had never received any antihyperglycemic treatment.

In this study, the status of depression was evaluated with the HADS-D. This scale was initially designed to identify depression in clinical psychiatric hospitals, yet it has also been adopted to screen depression in nonhospitalized population and considered to be accurate and convenient [32, 33]. It is a 7-item self-report questionnaire and each item is scored 0 to 3 and a total score of 8 or greater indicates the presence of depression. However it was reported that the best accuracy was achieved with cutoff of 10 points in total [32]. The internal consistency of HADS-D was assessed with Cronbach's alpha coefficient, which was 0.716 for T2D group and 0.730 for Ctrl group. It is regarded as satisfactory since the value of Cronbach's alpha is over 0.7 [18].

There are still some limitations in this study. Firstly, this is a cross-sectional study and the sample size was small. So we should draw the conclusion very carefully and prospective studies with large sample size are needed to verify the findings. Secondly, although only the newly diagnosed patients were enrolled in this study, the hyperglycemic status without obvious diabetic symptoms might exist earlier before the diabetes was diagnosed [34]; therefore the latent hyperglycemia and oxidative stress could take effect on telomere length for some time. There may be discrepancy of the duration in the diabetic subjects and it is hard to be evaluated. Thirdly, the chronic diabetic complications including diabetic nephropathy, retinopathy, and neuropathy have not been evaluated in this study yet, which might indicate the long duration of diabetes. Fourthly, there are still some other factors that can affect the development of depression which are not evaluated in this study, such as economic income and social status [35, 36].

## 5. Conclusion

In summary, this study indicated that oxidative stress contributes to both telomere length shortening and depression development in newly diagnosed type 2 diabetic patients. What is more, in depression status some other mechanisms besides oxidative stress may also affect the telomere length. To fully elucidate the complicated interactions of diabetes and depression with oxidative stress and cell senility, more research work is needed in the future.

## Figures and Tables

**Table 1:** Demographic, clinical, and biochemical parameters of the study subjects of T2D and Ctrl.

	T2D *n* = 71	Ctrl *n* = 52	*P*
Age (yrs)	54.55 ± 8.37	51.27 ± 7.66	0.028
Male [(*n* (%)]	40 (56.34)	30 (57.69)	0.881
Current smokers [(*n* (%)]	21 (29.58)	14 (26.92)	0.784
Current drinkers [(*n* (%)]	13 (18.31)	8 (15.38)	0.670
Use of aspirin [(*n* (%)]	8 (11.27)	4 (7.69)	0.509
Use of statin [(*n* (%)]	7 (9.86)	4 (7.69)	0.677
Use of antihypertensive drugs[(*n* (%)]	6 (8.45)	5 (9.62)	0.823
HADS-D score (point)*	8 (6, 9)	8 (6, 9)	0.200
HADS-D ≥ 10 [(*n* (%)]	17 (23.9)	5 (9.6)	0.041
BMI (kg/m^2^)	25.21 ± 2.19	23.86 ± 1.47	0.000
Waist circumstance (cm)	87.5 (81.8, 94.0)	82.0 (78.0, 86.0)	0.000
WHR	0.84 (0.77, 0.89)	0.80 (0.74, 0.84)	0.000
Systolic blood pressure (mmHg)	132 (120, 141)	127 (123, 130)	0.040
Diastolic blood pressure (mmHg)	81 (75, 88)	74 (68, 80)	0.000
HbA1c (%)	8.29 (7.58, 8.90)	5.10 (4.80, 5.40)	0.000
FPG (mmol/L)	8.72 ± 1.39	5.44 ± 0.35	0.000
FIN (mIU/L)	11.44 (6.99, 13.29)	12.22 (7.8, 14.32)	0.520
HOMA-IR	4.51 (2.73, 5.54)	2.93 (2.00, 3.29)	0.000
Total cholesterol (mmol/L)	4.69 ± 0.90	4.05 ± 0.54	0.000
Triglyceride (mmol/L)	1.86 (1.12, 2.31)	1.20 (0.86, 1.32)	0.000
HDL-cholesterol (mmol/L)	1.15 ± 0.24	1.22 ± 0.27	0.020
LDL-cholesterol (mmol/L)	2.47 ± 0.78	2.21 ± 0.67	0.050
8-OHdG (ng/mL)	1.75 ± 0.51	1.16 ± 0.31	0.000
T/S ratio	2.01 ± 0.47	2.28 ± 0.25	0.000

Data are means ± SD, *n* (%), or median (interquartile range).

T2D: type 2 diabetic group, Ctrl: control group, HADS-D: Depression Subscale of Hospital Anxiety and Depression Scale, BMI: body mass index, WHR: waist-to-hip ratio, FPG: fasting plasma glucose, FIN: fasting insulin, HOMA-IR: homeostasis model assessment-insulin resistance [HOMA-IR = FIN (mIU/L) × FPG (mmol/L)/22.5], 8-OHdG: Human 8-hydroxy-desoxyguanosine.

*The total score of HADS-D is 21 and depression was identified as the score ≥10.

**Table 2:** Demographic, clinical, and biochemical parameters of the study subjects of subgroups with/without depression in T2D^a^ and Ctrl^b^ groups.

	T2D		Ctrl		*F*	*P*
	D+	D−	D+	D−		
	*n* = 17	*n* = 54	*n* = 6	*n* = 46		
Age (yrs)	54.71 ± 8.10	54.83 ± 8.58	55.33 ± 6.56	51.22 ± 8.32	1.823	0.147
Male [(*n*(%)]	10 (58.82)	24 (44.44)	3 (50.00)	19 (41.30)	—	0.656
Current smokers [(*n*(%)]	5 (29.41)	16 (29.63)	2 (33.33)	12 (26.09)	—	0.971
Current drinkers [(*n*(%)]	2 (11.76)	11 (20.37)	1 (16.67)	7 (15.22)	—	0.834
Use of aspirin [(*n*(%)]	1 (5.88)	7 (12.96)	0 (0.00)	4 (8.70)	—	0.653
Use of statin [(*n*(%)]	3 (17.65)	4 (7.41)	1 (16.67)	3 (6.52)	—	0.474
Use of antihypertensive drugs [(*n*(%)]	3 (17.64)^#^	3 (5.56)^#^	0 (0.00)	5 (10.87)	—	0.00
HADS-D (point)	12 (10, 12)^∗⊙^	7 (6, 8)^#^	12 (10, 12)*	7 (6, 8)	—	0.000
BMI (kg/m^2^)	24.96 ± 1.93*	25.29 ± 2.28*	24.00 ± 2.32*	23.84 ± 1.36	5.033	0.003
Waist circumstance (cm)	87 (80, 91)*	88 (82, 94)*	81 (77, 87)	82 (78, 86)	—	0.000
WHR	0.82 (0.74, 0.88)	0.85 (0.79, 0.90)*	0.82 (0.75, 0.86)	0.80 (0.74, 0.84)	—	0.006
Systolic blood pressure (mmHg)	134 (120, 141)	131 (120, 141)	128 (123, 130)	126 (123, 130)	—	0.171
Diastolic blood pressure (mmHg)	79 (70, 84)	82 (75, 89)*	78 (72, 85)	74 (68, 131)	—	0.004
HbA1c (%)	8.4 (8, 9.1)^∗#^	8.2 (7.5, 8.7)^∗#^	5.3 (5.2, 5.5)	5.1 (4.7, 5.4)	—	0.000
FPG (mmol/L)	9.42 ± 1.66^∗#^	8.50 ± 1.23^∗#^	5.42 ± 0.28	5.44 ± 0.36	101.427	0.000
FIN (mIU/L)	12.55 (6.69, 15.02)	11.09 (6.99, 13.19)	12.95 (9.57, 16.40)	12.00 (7.74, 13.23)	—	0.617
HOMA-IR	5.54 (3.24, 6.25)*	4.19 (2.58, 5.02)*	3.09 (2.26, 3.70)	2.91 (2.00, 3.23)	—	0.000
Total cholesterol (mmol/L)	4.57 ± 1.03^∗#^	4.73 ± 0.87^∗#^	4.03 ± 0.26	4.06 ± 0.57	6.821	0.000
Triglyceride (mmol/L) HDL-cholesterol (mmol/L)	1.89 (1.34, 1.88)*	1.91 (1.08, 2.36)*	1.74 (1.18, 2.13)	1.23 (0.85, 1.55)	—	0.001
	1.15 ± 0.26	1.14 ± 0.23	1.20 ± 0.40	1.26 ± 0.25	1.841	0.143
LDL-cholesterol (mmol/L)	2.39 ± 0.92	2.50 ± 0.74	2.10 ± 0.81	2.22 ± 0.66	1.458	0.229
8-OHdG (ng/mL)	1.80 ± 0.42*	1.48 ± 0.44*	1.53 ± 0.26	1.09 ± 0.34	15.722	0.000
T/S ratio	1.70 (1.36, 2.06)^∗⊙^	2.11 (1.82, 2.42)*	2.01 (1.91, 2.13)*	2.32 (2.14, 2.47)	—	0.000

Data are means ± SD, *n* (%), or median (interquartile range).

T2D: type 2 diabetic group, Ctrl: control group, HADS-D: Depression Subscale of Hospital Anxiety and Depression Scale, BMI: body mass index, WHR: waist-to-hip ratio, FPG: fasting plasma glucose, FIN: fasting insulin, HOMA-IR: homeostasis model assessment-insulin resistance [HOMA - IR = FIN (mIU/L) × FPG (mmol/L)/22.5], 8-OHdG: Human 8-Hydroxy-desoxyguanosine.

^a^subgroup with/without depression in T2D is shown as T2D(D+)/T2D(D−), respectively,

^b^subgroup with/without depression in Ctrl group is shown as Ctrl(D+)/Ctrl(D−), respectively.

*P < 0.05 for T2D(D+)/T2D(D−)/Ctrl(D+) versus Ctrl(D−).

^#^ *P* < 0.05 for T2D(D+)/T2D(D−) versus Ctrl(D+).

^⊙^ *P* < 0.05 for T2D(D+) versus T2D(D−).

**Table 3:** Predictors of leukocyte telomere length in type 2 diabetic patients.

	Univariate analysis		Multiple linear regression	
	Unstandardized coefficient (*b*)	*P*	Unstandardized coefficient (*b*) (95% confidence interval for *b*)	*P*
Age	−0.027	0.000	−0.012 (−0.019, −0.005)	0.002
BMI	0.011	0.683
Waist circumference	−0.006	0.390
WHR	0.074	0.920
SBP	−0.005	0.138	−0.006 (−0.010, −0.003)	0.001
DBP	−0.002	0.748		
FPG	−0.204	0.000	−0.062 (−0.115, −0.009)	0.023
HbA1c	−0.287	0.000	−0.157 (−0.224, −0.090)	0.000
FIN	−0.003	0.744		
HOMA-IR	−0.026	0.135	0.016 (−0.003, 0.035)	0.097
Total cholesterol	−0.014	0.823
Triglyceride	−0.041	0.473
HDL	0.28	0.242
LDL	−0.071	0.326
HADS-D	−0.115	0.000	−0.057 (−0.080, −0.033)	0.000
8-OHdG	−0.599	0.000	−0.238 (−0.369, −0.103)	0.001

## Abbreviations

T2D: Type 2 diabetes
Ctrl: Control group
T/S ratio: Telomere length ratio
8-OHdG: Human 8-hydroxy-desoxyguanosine
HADS-D: The Depression Subscale of Hospital Anxiety and Depression Scale
HOMA-IR: Homeostasis model assessment-insulin resistance
ROS: Reactive oxygen species
FPG: Fasting plasma glucose
FIN: Fasting insulin
TC: Total cholesterol
TG: Triacylglycerols
HDL-C: High-density lipoprotein cholesterol
LDL-C: Low-density lipoprotein cholesterol
BMI: Body mass index
WHR: Waist-to-hip ratio.
